# Infant massage programs for newborn babies: systematic review

**DOI:** 10.1080/07853890.2021.1896600

**Published:** 2021-09-28

**Authors:** Sónia Vicente, Ângela Pereira

**Affiliations:** a Department of Physiotherapy, Escola Superior de Saúde Egas Moniz (ESSEM), Caparica, Portugal; b Centro de Investigação Interdisciplinar Egas Moniz (CiiEM), Egas Moniz Cooperativa de Ensino Superior, Caparica, Portugal; c Garcia de Orta Hospital, Almada, Portugal

## Abstract

**Introduction:**

Parenthood is a period of stress and great demand for parents. Taking care of a baby requires parental adjustment and behavioural development in order to satisfy child’s needs. Infant massage is an important parental support strategy which enhances parent-child relationship and promotes baby’s development [1]. This is a widely and effective technique used in preterm and term infants. In recent years, many studies showed several benefits, such as improved weight gain; pain reduction; relaxation; increased alertness and learning; decreased stress, depression and anxiety levels; promoted deep sleep and improved immune system [2–4]. However, when evaluating term massage programs, it is noticed that there are no similar methodologies between studies. The purpose of this study is to review massage programs for newborn babies.

**Materials and methods:**

This is a systematic review study. A literature search was conducted *via* three databases: PubMed, PEDro and Scielo using the search terms “Massage therapy”; “Infant massage”; “Baby massage”; “Full term”; “term babies”; “neonates”, “newborn” and “Maternal support” or “Mother support”. The inclusion criteria were: Studies published in English, Spanish or Portuguese; RCT studies; Quasi-experimental studies; Studies with massage program; Studies with term babies’ samples; Studies published between 2009 and 2019. The exclusion criteria were studies with term “babies with congenital disease”. A total of 62 papers were found and analysed by both authors. Fourteen met the criteria, 5 RCT’s and 9 quasi experimental studies.

**Results:**

Studies described 6 programs of infant massage to newborn babies. Ten studies described mothers applying term massage program, 3 applied by health professionals and 1 divided between health professionals when in hospital and by their mothers when babies were discharged. The most representative direction of massage was from head to feet. Majority of the studies used group strategy for teaching infant massage to mothers. Programs varied from 1–3 days twice a day for 15 mn to once a week between 30–60 mn during 4–8 weeks. Studies were scored by PEDro’s scale and ranged from 2 to 7. Half of the studies obtained score 5.

**Discussion and conclusion:**

We can conclude that 6 massage programs are described in literature; the majority is performed by babies’ mothers and there is a wide variety concerning program duration and frequency. Studies outcomes showed effects both on newborn babies and mother–baby relationship. Infant massage programs seem to be an important group teaching strategy for new parents. However, more studies should be done in order to understand if newborn massage works, regardless of the program type.
